# Wet Chemical Controllable Synthesis of Hematite Ellipsoids with Structurally Enhanced Visible Light Property

**DOI:** 10.1155/2013/410594

**Published:** 2013-10-03

**Authors:** Chengliang Han, Jie Han, Qiankun Li, Jingsong Xie

**Affiliations:** ^1^Department of Chemical and Material Engineering, Hefei University, Hefei 230601, China; ^2^Institute of Solid State Physics, Chinese Academy of Sciences, Hefei 230031, China

## Abstract

A facile and economic route has been presented for mass production of micro/nanostructured hematite microcrystals based on the wet chemical controllable method. The as-prepared samples were characterized using X-ray diffraction, scanning electron microscopy, transmission electron microscopy, and UV-Vis absorption spectroscopy. The results showed that the product was mesoporous **α**-Fe_2_O_3_ and nearly elliptical in shape. Each hematite ellipsoid was packed by many **α**-Fe_2_O_3_ nanoparticles. The values of vapor pressure in reaction systems played vital roles in the formation of porous hematite ellipsoids. Optical tests demonstrated that the micro/nanostructured elliptical hematite exhibited enhanced visible light property at room temperature. The formation of these porous hematite ellipsoids could be attributed to the vapor pressure induced oriented assembling of lots of **α**-Fe_2_O_3_ nanoparticles.

## 1. Introduction

Hematite (*α*-Fe_2_O_3_) is the oldest known iron oxide mineral and is widely applied in catalysts, gas sensors, pigments, and promising photoanodes for solar cells [1–4]. Over the past ten years, various hematite nanostructures with a well-defined shape such as nanorods [5], nanowires [6], and nanobelts [7] have been obtained successfully by directional growth techniques [8–10], template guiding [11, 12], and decomposition of shape-regular iron precursors [13, 14]. However, the previous reported shape-controllable synthetic methods have some limitations. For example, the used shape-controlling reagents such as some templates and surfactants are usually more expensive and hard to wash out. Additionally, solid reaction processes for *α*-Fe_2_O_3_ may introduce some other iron oxide phases and release harmful gases for possible environmental pollution. Consequently, developing simple and economic methods for preparation of hematite nanomaterials as well as the modification of their sizes, morphology, and porosity has been intensively pursued not only for their fundamental scientific interest but also for many technological applications. This work presents a new method to produce large-scale *α*-Fe_2_O_3_ micro/nanostructured porous ellipsoids. The size and shape of as-prepared *α*-Fe_2_O_3_ can be well controlled only by simply regulating the values of vapor pressure in reaction systems. The results reported in this paper mainly encompass the formation mechanism of porous *α*-Fe_2_O_3_ with different morphologies and their corresponding visible light properties at room temperature.

## 2. Materials and Methods

### 2.1. Preparation of the Samples

The micro/nanostructured hematites were prepared as follows. Firstly, the Fe(OH)_3_ precursors were precipitated from FeCl_3_ *·*6H_2_O solution by adding proper ammonia (NH_3_ *·*H_2_O). And the above Fe(OH)_3_ with the solution was transferred into a 100 mL Teflon autoclave with a pressure gage for detecting internal vapor pressure. Then, the above sealed Teflon autoclave was heated to 453 K, and the internal vapor pressure was kept under 1.45 × 10^5^ Pa for 2 h. After cooling to ambient temperature, the bright red powder was ultrasonically rinsed for several times in deionized water and ethanol, respectively. Finally, the sample was collected by a centrifuge and dried in a vacuum oven at 353 K for 6 h.

### 2.2. Characterization of the Samples

X-ray powder diffraction (XRD) patterns were recorded on a Philips X'pert diffractometer using CuK_*α*_ radiation (*λ* = 1.5419 Å). Scanning electron microscopy (SEM) was recorded on a Sirion 200 FEI field emission scanning electron microscope. The transmission electron microscopic (TEM) micrographs were taken with a JEOL-2010 field emission transmission electron microscope with an accelerating voltage of 200 kV. The UV-Vis absorption spectra were taken on a spectrophotometer (Cary 5E UV-Vis-NIR) from 200 to 1200 nm.

## 3. Results and Discussion

### 3.1. XRD and Morphology of As-Prepared Samples

**Figure 1:**
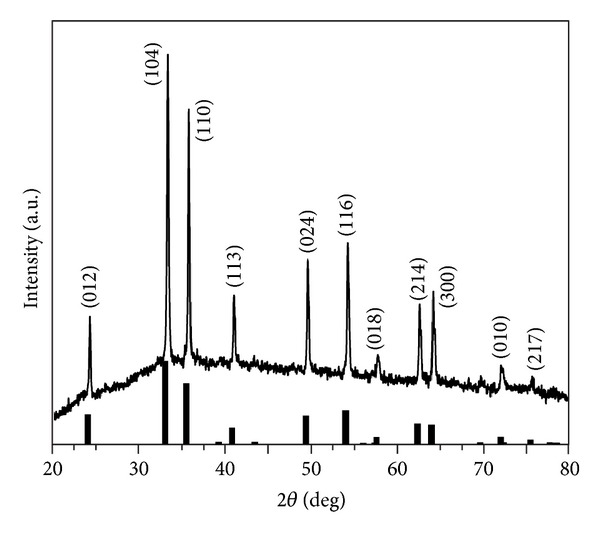
XRD patterns of the as-synthesized sample and standard *α*-Fe_2_O_3_ powder (the line spectrum).

After Fe(OH)_3_ precursors in solution were heated at 453 K for 2 h in Teflon autoclave with the internal vapor 1.45 × 10^5^ Pa or so, the red powders were collected by a centrifuge. The corresponding X-ray diffraction (XRD) was illustrated in Figure 1. It can be found that all the diffraction peaks can be indexed to pure *α*-Fe_2_O_3_ (JCPDS NO. 89-2810). The intense peaks of the XRD pattern indicate that the as-prepared powders were well-crystallized *α*-Fe_2_O_3_.

**Figure 2:**
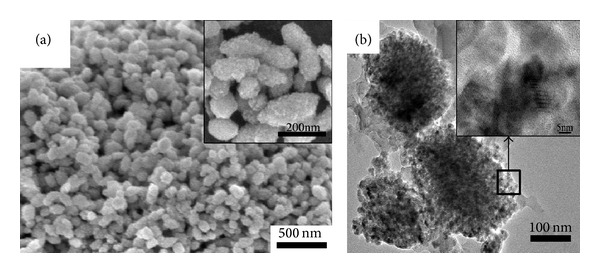
Microstructural examination of *α*-Fe_2_O_3_ ellipsoids. (a) Low-magnification SEM image; (b) low-magnification TEM image (insets: corresponding high-magnification).

FE-SEM observations have shown that each as-prepared *α*-Fe_2_O_3_ particle is of collective-like morphology with nearly elliptical in shape and nanoscaled surface roughness, as illustrated in Figure 2(a) and its inset. Each hematite ellipsoid is 500 nm and 120 nm in major and short axis, respectively (seen from Figure 2(a)). Further microstructural examination conducted for such *α*-Fe_2_O_3_ ellipsoids was shown in Figure 2(b). It was confirmed that many *α*-Fe_2_O_3_ nanoparticles (~20 nm) reconstructed the porous hematite ellipsoid. The surface of every ellipsoid is also thus rough.

**Figure 3:**
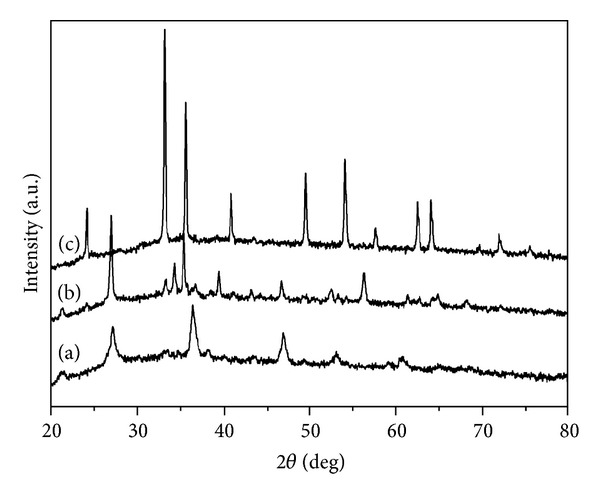
XRD patterns of samples under different pressures ((a) 1.02 × 10^5^ Pa (b) 1.15 × 10^5^ Pa (c) 1.62 × 10^5^ Pa).

**Figure 4:**
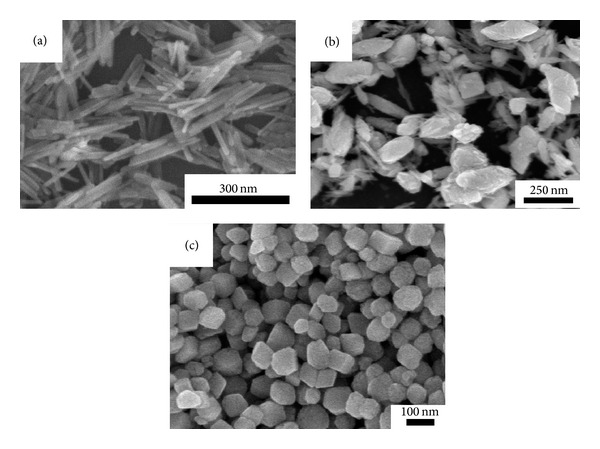
Morphology of samples at different pressures ((a) 1.02 × 10^5^ Pa, (b) 1.15 × 10^5^ Pa, and (c) 1.62 × 10^5^ Pa).

In general, the values of pressure in reaction systems will determine the chemical reaction start at the same reaction temperature. So different composition products can be gained under different values of pressures. Our deep and systematic studies have revealed that pure goethite (*α*-FeOOH) nanorods (seen from Figures 3(a) and 4(a)) will be obtained when the value of reactive pressure is less than 1.02 × 10^5^ Pa. And the mixture of *α*-FeOOH nanorods and *α*-Fe_2_O_3_ ellipsoids will be acquired when the value of pressure is between 1.15 × 10^5^ Pa and 1.45 × 10^5^ Pa, as shown in Figures 3(b) and 4(b), respectively. 

### 3.2. Effect of Vapor Pressure

When the value of pressure is next to 1.62 × 10^5^ Pa or higher, pure *α*-Fe_2_O_3_ nanocrystals will be achieved (seen from Figure 3(c)). Hence, a lower value of reactive pressure was not beneficial to the formation of *α*-Fe_2_O_3_. However, higher value of pressure will lead to lots of homogeneous *α*-Fe_2_O_3_ solid quasicubic particles (seen from Figure 4(c)) instead of the porous *α*-Fe_2_O_3_ ellipsoids, as illustrated in Figure 2. Therefore, it can be concluded that the values of vapor pressure in reaction systems will influence not only the composition of the products but also the morphologies.

### 3.3. Formation of Porous *α*-Fe_2_O_3_ Ellipsoids

The formation process of *α*-Fe_2_O_3_ porous ellipsoids could be described in the following three stages. First of all, the Fe(OH)_3_ precursors would be formed by the precipitation between Fe^3+^ and OH^−^ at room temperature (1). With subsequent heating at 453 K, the Fe(OH)_3_ will begin to decompose into *α*-FeOOH (2), which is finally dehydrated to *α*-Fe_2_O_3_ molecules (3). Secondly, when the concentration of *α*-Fe_2_O_3_ molecules was supersaturated, ultrafine *α*-Fe_2_O_3_ particles would be formed in the solution by nucleation and growth:

(1) $$\begin{array}{c} \text{F}{\text{e}}^{3+}+3\text{O}{\text{H}}^{-}\overset{298\;\text{K}}{\to }\text{Fe}{\left(\text{OH}\right)}_{3} \end{array}$$

(2) $$\begin{array}{c} \text{Fe}{\left(\text{OH}\right)}_{3}\overset{453\;\text{K}}{\to }\alpha \text{-FeOOH}+{\text{H}}_{2}\text{O} \end{array}$$

(3) $$\begin{array}{c} 2\alpha \text{-FeOOH}\overset{453\;\text{K}}{\to }\alpha \text{-F}{\text{e}}_{2}{\text{O}}_{3}+2{\text{H}}_{2}\text{O} \end{array}$$

**Figure 5:**
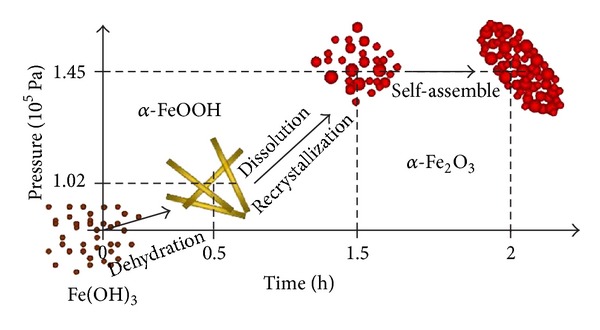
Schematic illustration for formation of porous *α*-Fe_2_O_3_ ellipsoids.

Finally, the as-formed hematite nanoparticles would be further coarsened by an oriented-assembling mode in order to reduce the surface energy of *α*-Fe_2_O_3_ particles which were polar crystals and tended to spontaneously assemble [15, 16]. The proper value of vapor pressure will help *α*-Fe_2_O_3_ nanocrystals migrate and readjust more easily in a reactor. As a result, with the assemble going on, the porous hematite ellipsoid packed with many *α*-Fe_2_O_3_ nanoparticles would be formed. The above three stages have been illustrated in Figure 5. We acknowledge that the true formation mechanism of porous *α*-Fe_2_O_3_ elliptical structures is still unclear. However, it is obvious that the values of vapor pressure in a reactor are of great importance in the growth of porous *α*-Fe_2_O_3_ ellipsoids. 

### 3.4. Ultraviolet Visible Optical Properties

The optical absorption measurement of as-prepared *α*-Fe_2_O_3_ products was conducted at room temperature, which may help the understanding of the electronic structure and size effect.

**Figure 6:**
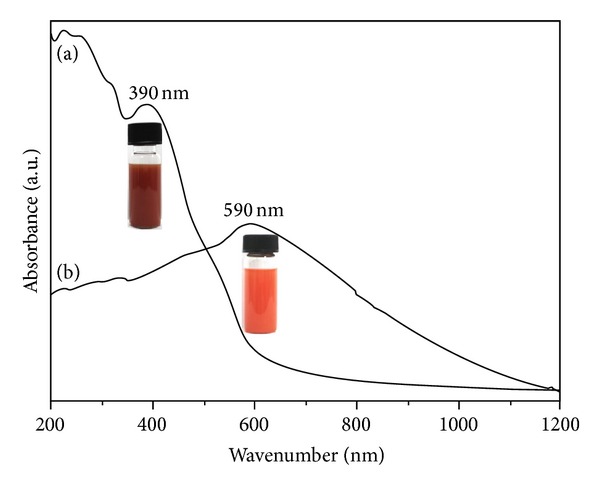
UV-Vis spectrums of *α*-Fe_2_O_3_ from various pressures: (a) 1.62 × 10^5^ Pa; (b) 1.45 × 10^5^ Pa.

Figure 6(a) shows the optical absorption spectrum of the quasicubic *α*-Fe_2_O_3_ nanostructures, and the optical absorption feature was observed at wavelength around 390 nm (3.18 eV) and had an inflection and shift to the short wavelength compared to that reported by Li et al. [17]. Figure 6(b) shows the optical absorption spectrum of the ellipsoid-like micro/nanostructured *α*-Fe_2_O_3_, and the absorption peak is about 590 nm (2.10 eV). The different optical adsorption properties of two different shaped *α*-Fe_2_O_3_ can be attributed to their different structures. In a word, the as-obtained porous *α*-Fe_2_O_3_ ellipsoids were extended for potential application in photodegradating pollutants under visible light. The relative studies are our next work.

## 4. Conclusions

In conclusion, we have demonstrated mass production of micro/nanostructured elliptical hematite by a wet chemical pressure-controlled method. Our investigations have showed that the values of vapor pressure in reaction systems have been considered to play vital roles in the formation of porous *α*-Fe_2_O_3_ ellipsoids. The vapor pressure induced oriented assembling mechanism of polar hematite nanocrystals has been inferred. Importantly, the micro/nanostructured porous *α*-Fe_2_O_3_ ellipsoids with excellent visible light property can be used as a novel potential photocatalyst for removal of some toxic chemicals.
